# A healthy heart is not a metronome: an integrative review of the heart's anatomy and heart rate variability

**DOI:** 10.3389/fpsyg.2014.01040

**Published:** 2014-09-30

**Authors:** Fred Shaffer, Rollin McCraty, Christopher L. Zerr

**Affiliations:** ^1^Center for Applied Psychophysiology, Department of Psychology, Truman State UniversityKirksville, MO, USA; ^2^HeartMath Research Center, Institute of HeartMathBoulder Creek, CA, USA

**Keywords:** heart rate variability, psychophysiological coherence, neurocardiology, biofeedback interventions, emotional self-regulation

## Abstract

Heart rate variability (HRV), the change in the time intervals between adjacent heartbeats, is an emergent property of interdependent regulatory systems that operate on different time scales to adapt to challenges and achieve optimal performance. This article briefly reviews neural regulation of the heart, and its basic anatomy, the cardiac cycle, and the sinoatrial and atrioventricular pacemakers. The cardiovascular regulation center in the medulla integrates sensory information and input from higher brain centers, and afferent cardiovascular system inputs to adjust heart rate and blood pressure via sympathetic and parasympathetic efferent pathways. This article reviews sympathetic and parasympathetic influences on the heart, and examines the interpretation of HRV and the association between reduced HRV, risk of disease and mortality, and the loss of regulatory capacity. This article also discusses the intrinsic cardiac nervous system and the heart-brain connection, through which afferent information can influence activity in the subcortical and frontocortical areas, and motor cortex. It also considers new perspectives on the putative underlying physiological mechanisms and properties of the ultra-low-frequency (ULF), very-low-frequency (VLF), low-frequency (LF), and high-frequency (HF) bands. Additionally, it reviews the most common time and frequency domain measurements as well as standardized data collection protocols. In its final section, this article integrates Porges' polyvagal theory, Thayer and colleagues' neurovisceral integration model, Lehrer et al.'s resonance frequency model, and the Institute of HeartMath's coherence model. The authors conclude that a coherent heart is not a metronome because its rhythms are characterized by both complexity and stability over longer time scales. Future research should expand understanding of how the heart and its intrinsic nervous system influence the brain.

## Introduction

### The heart

The heart is about the size of a closed fist, weighs between 250 and 350 g, and beats approximately 100,000 times a day and 2.5 billion times during an average lifetime. The muscular heart consists of two atria and two ventricles. The atria are upper receiving chambers for returning venous blood. The ventricles comprise most of the heart's volume, lie below the atria, and pump blood from the heart into the lungs and arteries. Deoxygenated blood enters the right atrium, flows into the right ventricle, and is pumped to the lungs via the pulmonary arteries, where wastes are removed and oxygen is replaced. Oxygenated blood is transported through the pulmonary veins to the left atrium and enters the left ventricle. When the left ventricle contracts, blood is ejected through the aorta to the arterial system (Marieb and Hoehn, 2013; Tortora and Derrickson, 2014).

### The cardiac cycle

The cardiac cycle consists of systole (ventricular contraction) and diastole (ventricular relaxation). During systole, blood pressure (BP) peaks as contraction by the left ventricle ejects blood from the heart. Systolic BP is measured during this phase. During diastole, BP is lowest when the left ventricle relaxes. Diastolic BP is measured at this time.

### Pacemakers

The heart contains autorhythmic cells that spontaneously generate the pacemaker potentials that initiate cardiac contractions. These cells continue to initiate heartbeats after surgeons sever all efferent cardiac nerves and remove a heart from the chest cavity for transplantation. Autorhythmic cells function as pacemakers and provide a conduction pathway for pacemaker potentials.

**Figure 1:**
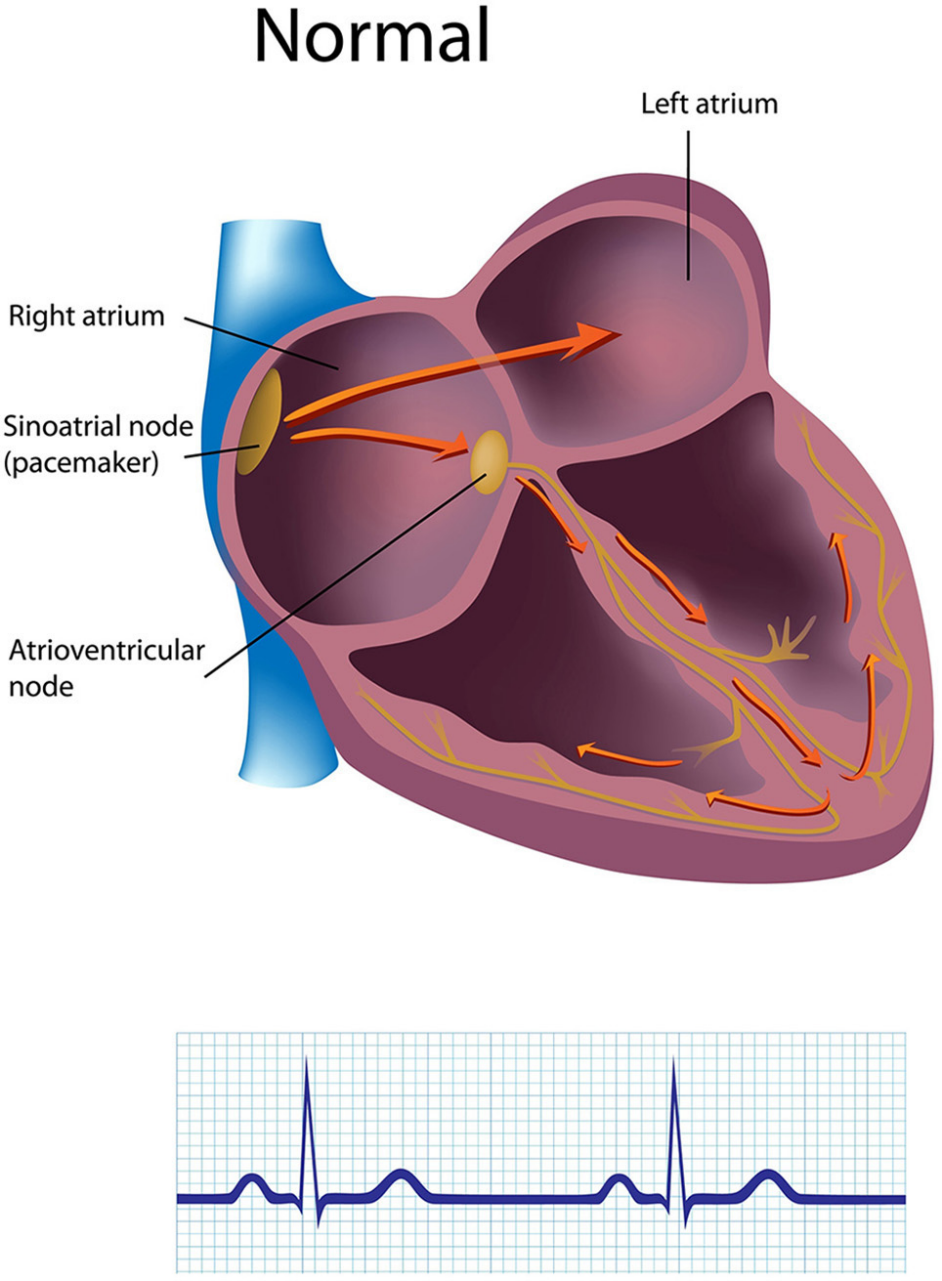
**The generation of the electrocardiogram**. Credit: Alila Sao Mai/Shutterstock.com.

The sinoatrial (SA) node and atrioventricular (AV) node are the two internal pacemakers that are primarily responsible for initiating the heartbeat. The electrocardiogram (ECG) records the action of this electrical conduction system and contraction of the myocardium (Figure 1).

### Cardiac conduction

**Figure 2:**
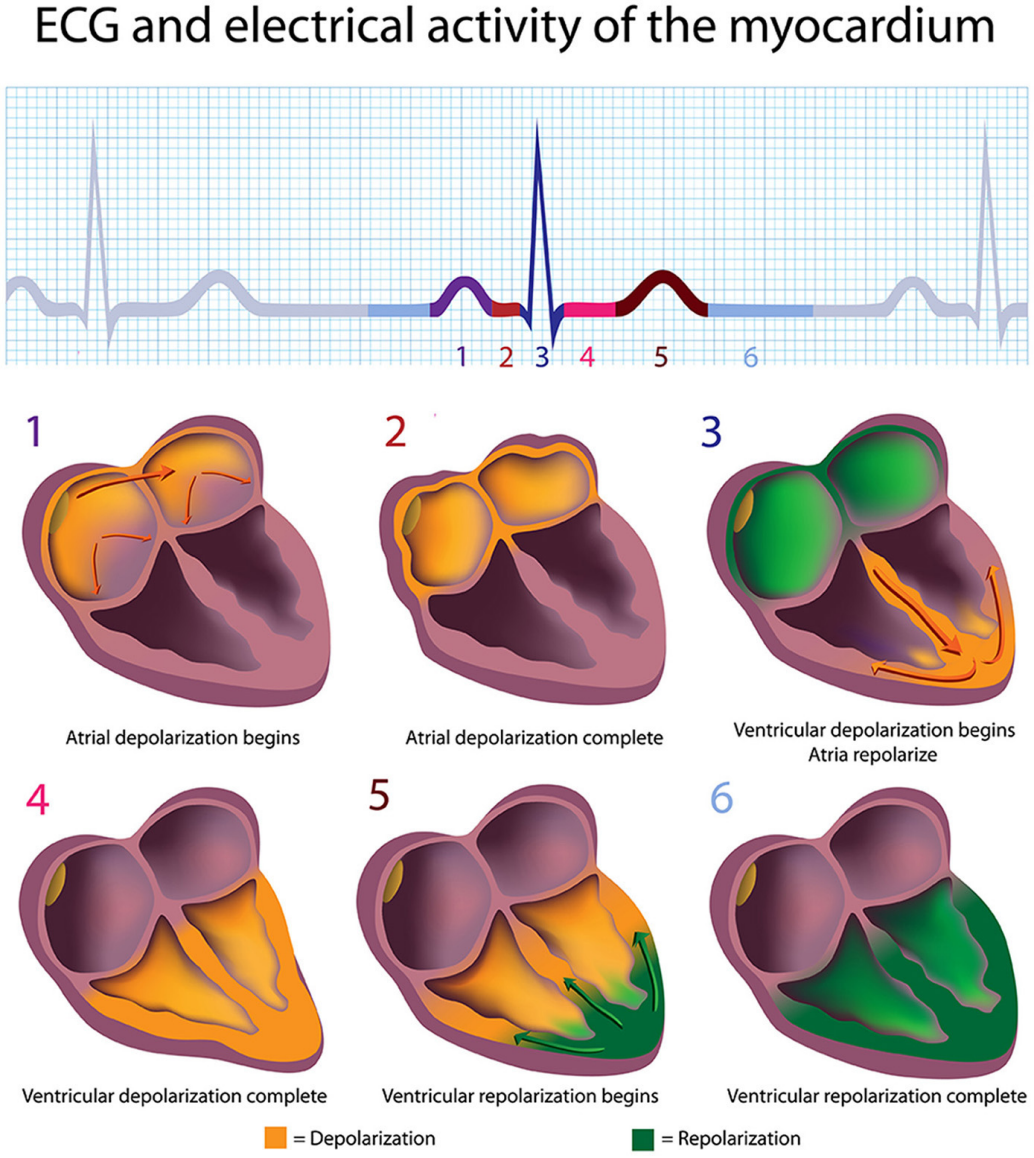
**The depolarization and repolarization of the heart**. Credit: Alila Sao Mai/Shutterstock.com.

In a healthy heart, the SA node initiates each cardiac cycle through spontaneous depolarization of its autorhythmic fibers. The SA node's intrinsic firing rate of about 60–100 action potentials per minute usually prevents slower parts of the conduction system and myocardium (heart muscle) from generating competing potentials. The AV node can replace an injured or diseased SA node as pacemaker and spontaneously depolarizes 40–60 times per minute. The SA node generates an electrical impulse that travels through the atria to the AV node in about 0.03 s and causes the AV node to fire (Figure 2). The P wave of the ECG is produced as muscle cells in the atria depolarize and culminates in contraction of the atria (atrial systole).

The signal rapidly spreads through the AV bundle reaching the top of the septum. These fibers descend down both sides of the septum as the right and left bundle branches and conduct the action potential over the ventricles about 0.2 s after the appearance of the P wave. Conduction myofibers, which extend from the bundle branches into the myocardium, depolarize contractile fibers in the ventricles (lower chambers), resulting in the QRS complex followed by the S-T segment. Ventricular contraction (ventricular systole) occurs after the onset of the QRS complex and extends into the S-T segment. The repolarization of ventricular myocardium generates the T wave about 0.4 s following the P wave. The ventricles relax (ventricular diastole) 0.6 s after the P wave begins (Tortora and Derrickson, 2014).

### Regulation of the heart

In a healthy organism, there is a dynamic relative balance between the sympathetic nervous system (SNS) and parasympathetic nervous system (PNS). PNS activity predominates at rest, resulting in an average HR of 75 beats per minute (bpm). This is significantly slower than the SA node's intrinsic rate, which decreases with age from an average 107 bpm at 20 years to 90 bpm at 50 years (Opthof, 2000). The parasympathetic branch can slow the heart to 20 or 30 bpm or briefly stop it (Tortora and Derrickson, 2014). This illustrates the response called accentuated antagonism (Olshansky et al., 2008). Parasympathetic nerves exert their effects more rapidly (<1 s) than sympathetic nerves (>5 s; Nunan et al., 2010).

A major cardiovascular center, located in the medulla of the brainstem, integrates sensory information from proprioceptors (limb position), chemoreceptors (blood chemistry), and mechanoreceptors (also called baroreceptors) from the heart and information from the cerebral cortex and limbic system. The cardiovascular center responds to sensory and higher brain center input by adjusting heart rate via shifts in the relative balance between sympathetic and parasympathetic outflow (Shaffer and Venner, 2013).

In a healthy individual, the HR estimated at any given time represents the net effect of the neural output of the parasympathetic (vagus) nerves, which slow HR, and the sympathetic nerves, which accelerate it. At rest, both sympathetic and parasympathetic nerves are tonically active with the vagal effects dominant. Therefore, HR reflects the relative activity of the sympathetic and parasympathetic systems; with the more important question being, is the relative balance (HR) appropriate for the context the person is engaged in at any given moment? In other words, is HR higher during the daytime and when dealing with challenging tasks, and lower at night, during sleep or when not engaged in challenging duties or activities?

The most obvious effect of vagal activity is to slow or even stop the heart. The vagus nerves are the primary nerves for the parasympathetic system and innervate the intrinsic cardiac nervous system and project to the SA node, AV node, and atrial cardiac muscle. Increased efferent activity in these nerves triggers acetylcholine release and binding to muscarinic (mainly M2) receptors. This decreases the rate of spontaneous depolarization in the SA and AV nodes, slowing HR. Because there is sparse vagal innervation of the ventricles, vagal activity minimally affects ventricular contractility.

The response time of the sinus node is very short and the effect of a single efferent vagal impulse depends on the phase of the cardiac cycle at which it is received. Thus, vagal stimulation results in an immediate response that typically occurs within the cardiac cycle in which it occurs and affects only one or two heartbeats after its onset. After cessation of vagal stimulation, HR rapidly returns to its previous level. An increase in HR can also be achieved by reduced vagal activity or vagal block. Thus, sudden changes in HR (up or down) between one beat and the next are parasympathetically mediated (Hainsworth, 1995).

An increase in sympathetic activity is the principal method used to increase HR above the intrinsic level generated by the SA node. Following the onset of sympathetic stimulation, there is a delay of up to 5 s before the stimulation induces a progressive increase in HR, which reaches a steady level in 20–30 s if the stimulus is continuous (Hainsworth, 1995). The slowness of the response to sympathetic stimulation is in direct contrast to vagal stimulation, which is almost instantaneous. However, the effect on HR is longer lasting and even a short stimulus can affect HR for 5–10 s. Efferent sympathetic nerves target the SA node and AV node via the intrinsic cardiac nervous system, and the bulk of the myocardium (heart muscle). Action potentials conducted by these motor neurons trigger norepinephrine (NE) and epinephrine (E) release and binding to beta-adrenergic (β 1) receptors located on cardiac muscle fibers. This speeds up spontaneous depolarization in the SA and AV nodes, increases HR, and strengthens the contractility of the atria and ventricles. In failing hearts, the number of β 1 receptors is reduced and their cardiac muscle contraction in response to NE and E binding is weakened (Ogletree-Hughes et al., 2001).

## Afferent modulation of cardiac and brain activity

**Figure 3:**
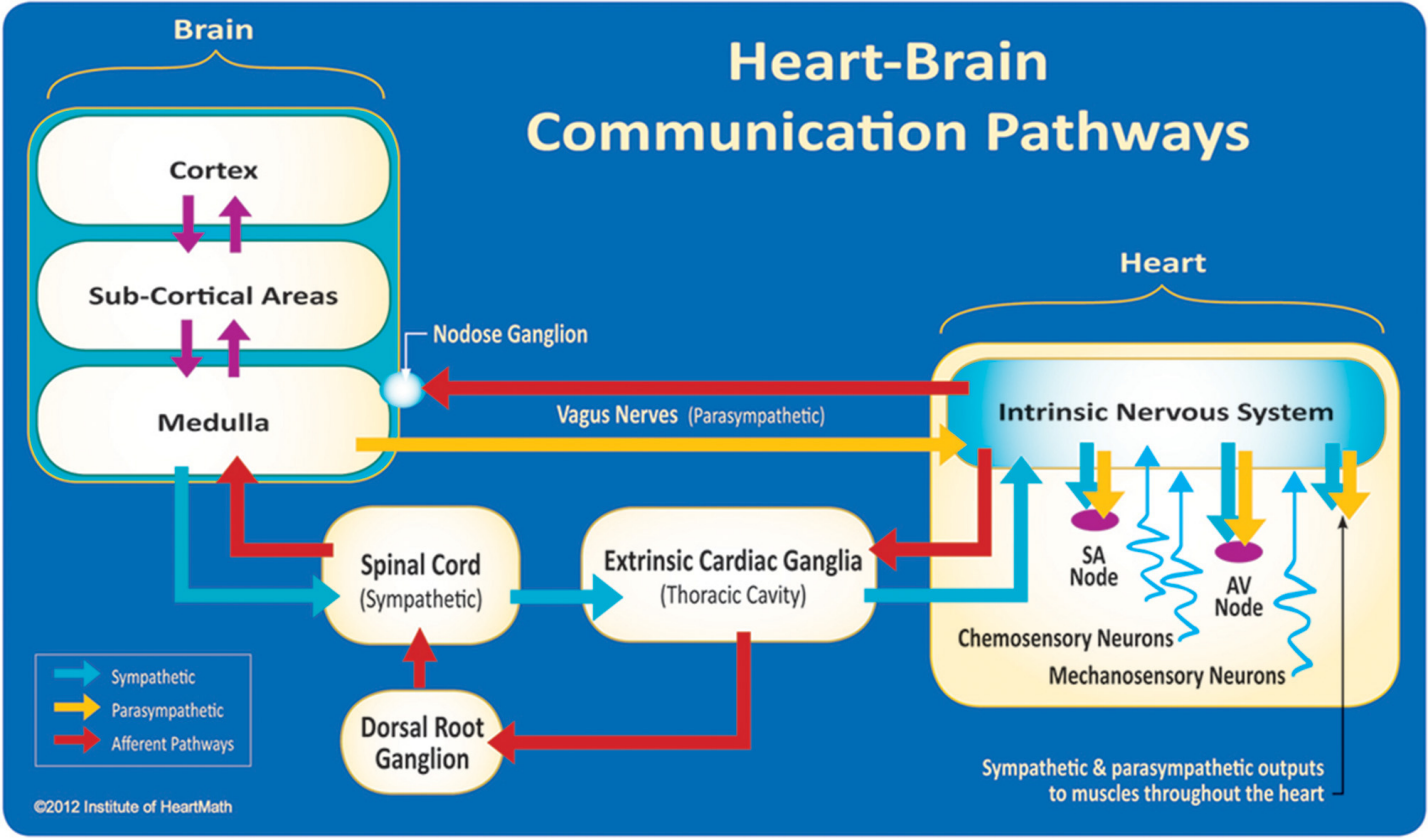
**The neural communication pathways interacting between the heart and the brain are responsible for the generation of HRV**. The intrinsic cardiac nervous system integrates information from the extrinsic nervous system and from the sensory neurites within the heart. The extrinsic cardiac ganglia located in the thoracic cavity have connections to the lungs and esophagus and are indirectly connected via the spinal cord to many other organs such as the skin and arteries. The vagus nerve (parasympathetic) primarily consists of afferent (flowing to the brain) fibers which connect to the medulla, after passing through the nodose ganglion. Credit: Institute of HeartMath.

The field of *neurocardiology* explores the anatomy and functions of the connections between the heart and brain (Davis and Natelson, 1993; Armour, 2003) and represents the intersection of neurology and cardiology. While efferent (descending) regulation of the heart by the autonomic nervous system (ANS) is well known, newer data have suggested a more complex modulation of heart function by the intrinsic cardiac nervous system (Kukanova and Mravec, 2006). These intracardiac neurons (sensory, interconnecting, afferent, and motor neurons) (Verkerk et al., 2012) can operate independently and their network is sufficiently extensive to be characterized as its own “little brain” on the mammalian heart (Armour, 2008, p. 165). The afferent (ascending) nerves play a critical role in physiological regulation and affect the heart's rhythm. Efferent sympathetic and parasympathetic activity are integrated with the activity occurring in the heart's intrinsic nervous system, including the afferent signals occurring from the mechanosensory and chemosensory neurons (Figure 3).

Interestingly, the majority of fibers in the vagus nerve (approximately 85–90%) are afferents, and signals are sent to the brain via cardiovascular afferents to a greater extent than by any other major organ (Cameron, 2002). Mechanical and hormonal information is transduced into neurological impulses by sensory neurons in the heart before being processed in the intrinsic nervous system. These impulses then travel to the brain via afferent pathways in the spinal column and vagus nerve (McCraty, 2011).

**Figure 4:**
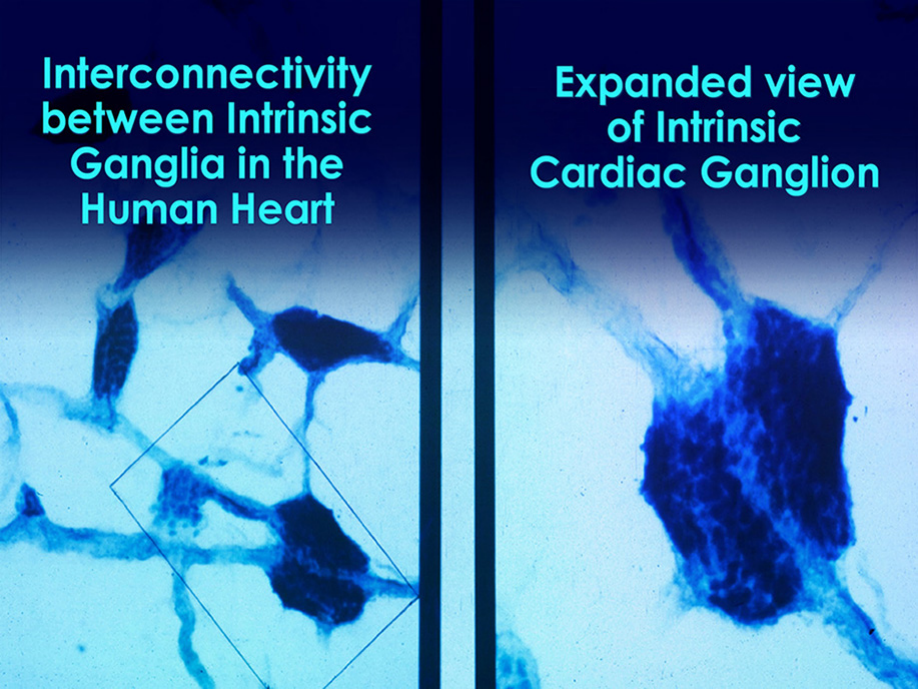
**Microscopic image of interconnected intrinsic cardiac ganglia in the human heart**. The thin, light blue structures are multiple axons that connect the ganglia. Credit: Dr. Andrew Armour and the Institute of HeartMath.

**Figure 5:**
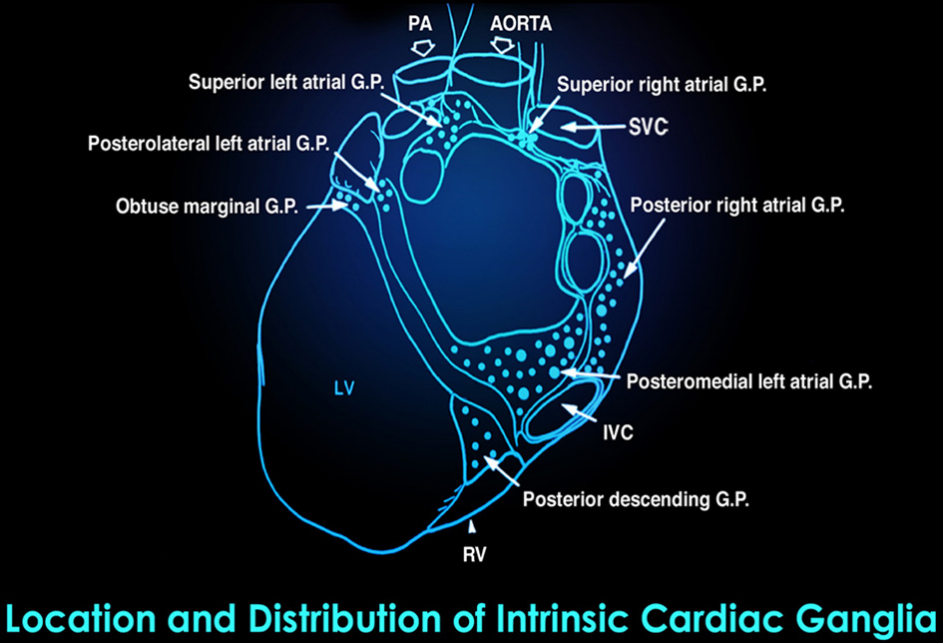
**This drawing shows the location and distribution of intrinsic cardiac ganglia which are interconnected and form the “heart brain.”** Note how they are distributed around the orifices of the major vessels. Credit: Dr. Andrew Armour and the Institute of HeartMath.

Short-term regulation of BP is accomplished by a complex network of pressure-sensitive baroreceptors or mechanosensitive neurons which are located throughout the heart and in the aortic arch. Since BP regulation is a central role of the cardiovascular system, the factors that alter BP also affect fluctuations in HR. Intrinsic cardiac afferent sensory neurons (Figures 4, 5) transduce and distribute mechanical and chemical information regarding the heart (Cheng et al., 1997) to the intrinsic cardiac nervous system (Ardell et al., 1991). The afferent impulses from the mechanosensitive neurons travel via the glossopharyngeal and vagal nerves to the nucleus of the solitary tract (NST), which connects with the other regulatory centers in the medulla to modulate SNS outflow to the heart and the blood vessels. There is also some modulation of parasympathetic outflow to the heart via connections to the dorsal vagal complex. Thus, mechanosensitive neurons affect HR, vasoconstriction, venoconstriction, and cardiac contractility in order to regulate BP (Hainsworth, 1995). This input from the heart can also modulate and impact hormonal release (Randall et al., 1981).

The heart not only functions as an intricate information processing and encoding center (Armour and Kember, 2004), but is also an endocrine gland that can produce and secrete its own hormones and neurotransmitters (Cantin and Genest, 1985, 1986; Mukoyama et al., 1991; Huang et al., 1996). For instance, atrial myocytes can secrete atrial natriuretic peptide (ANP), a hormone that promotes salt and water excretion, to lower BP and produce vasodilation (Dietz, 2005). Additionally, intrinsic cardiac adrenergic cells can synthesize and secrete catecholamines such as dopamine, NE, and E (Huang et al., 1996) in addition to high concentrations of oxytocin (Gutkowska et al., 2000).

Research insights from the field of neurocardiology have confirmed that the neural interactions between the heart and brain are more complex than thought in the past. This research has shown that complex patterns of cardiovascular afferent activity occur across time scales from milliseconds to minutes (Armour and Kember, 2004). This work has also shown that the intrinsic cardiac nervous system has both short-term and long-term memory functions, which can influence HRV and afferent activity related to pressure, rhythm, and rate, as well as afferent activity associated with hormonal factors (Armour, 2003; Armour and Kember, 2004; Ardell et al., 2009).

John and Beatrice Lacey conducted heart–brain interaction studies and were the first to suggest a causal role of the heart in modulating cognitive functions such as sensory-motor and perceptual performance (Lacey, 1967; Lacey and Lacey, 1970, 1974). They suggested that cortical functions are modulated via afferent input from pressure-sensitive neurons in the heart, carotid arteries, and aortic arch (Lacey, 1967). Their research focused on activity occurring within a single cardiac cycle, and they confirmed that cardiovascular activity influences perception and cognitive performance. Research by Velden and Wölk found that cognitive performance fluctuates at a rhythm around 10 Hz. They also demonstrated that the modulation of cortical function via the heart's influence is due to afferent inputs on the neurons in the thalamus which globally synchronizes cortical activity (Velden and Wölk, 1987; Wölk and Velden, 1989). An important aspect of their work was the finding that it is the “pattern and stability” (the rhythm) of the heart's afferent inputs, rather than the number of neural bursts within the cardiac cycle, that are important in modulating thalamic activity, which in turn has global effects on brain function.

This growing body of research indicates that afferent information processed by this intrinsic cardiac nervous system (Armour, 1991) can influence activity in the frontocortical areas (Lane et al., 2001; McCraty et al., 2004) and motor cortex (Svensson and Thorén, 1979), affecting psychological factors such as attention level, motivation (Schandry and Montoya, 1996), perceptual sensitivity (Montoya et al., 1993), and emotional processing (Zhang et al., 1986). Intrinsic cardiac afferent neurons project to nodose and dorsal root ganglia, the brainstem (dorsal root ganglia first project to the spinal cord), the hypothalamus, thalamus, or amygdala, and then to the cerebral cortex (Kukanova and Mravec, 2006; McCraty et al., 2009).

### Heartbeat evoked potentials

Heartbeat evoked potentials (HEPs) can be used to identify the specific pathways and influence of afferent input from the heart to the brain. HEPs are segments of electroencephalogram (EEG) that are synchronized to the heartbeat. The ECG R-wave is used as a timing source for signal averaging, resulting in waveforms known as HEPs. Changes in these evoked potentials associated with the heart's afferent neurological input to the brain are detectable between 50 and 550 ms after each heartbeat. There is a replicable and complex distribution of HEPs across the scalp. Researchers can use the location and timing of the various components of HEP waveforms, as well as changes in their amplitudes and morphology, to track the flow and timing of cardiovascular afferent information throughout the brain (Schandry and Montoya, 1996).

MacKinnon et al. (2013) reported that HRV influences the amplitude of heartbeat evoked potentials (HEP N250s). In this specific context, self-induction of either negative or positive emotion conditions by recalling past events reduced HRV and N250 amplitude. In contrast, resonance frequency breathing (breathing at a rate that maximizes HRV amplitude) increased HRV and HRV coherence (auto-coherence and sinusoidal pattern) above baseline and increased N250 amplitude. The authors speculated that resonance frequency breathing reduces interference with afferent signal transmission from the heart to the cerebral cortex.

## What is heart rate variability?

Ever since Walter Cannon introduced the concept of homeostasis in 1929, the study of physiology has been based on the principle that all cells, tissues, and organs maintain a static or constant “steady-state” condition in their internal environment. However, with the introduction of signal processing techniques that can acquire continuous time series data from physiologic processes such as heart rate, BP, and nerve activity, it has become abundantly apparent that biological processes vary in a complex and nonlinear way, even during “steady-state” conditions. These observations have led to the understanding that healthy physiologic function is a result of continuous, dynamic interactions between multiple neural, hormonal, and mechanical control systems at both local and central levels. For example, we now know that the normal resting sinus rhythm of the heart is highly irregular during steady-state conditions rather than being monotonously regular, which was the widespread notion for many years. *A healthy heart is not a metronome*.

With the ability to measure the ECG in 1895, and the later development of modern signal processing which first emerged in the 1960s and 1970s, the investigation of the heart's complex rhythm rapidly exploded. The irregular behavior of the heartbeat is readily apparent when heart rate is examined on a beat-to-beat basis, but is overlooked when a mean value over time is calculated. These fluctuations in heart rate result from complex, non-linear interactions between a number of different physiological systems (Reyes Del Paso et al., 2013).

**Figure 6:**
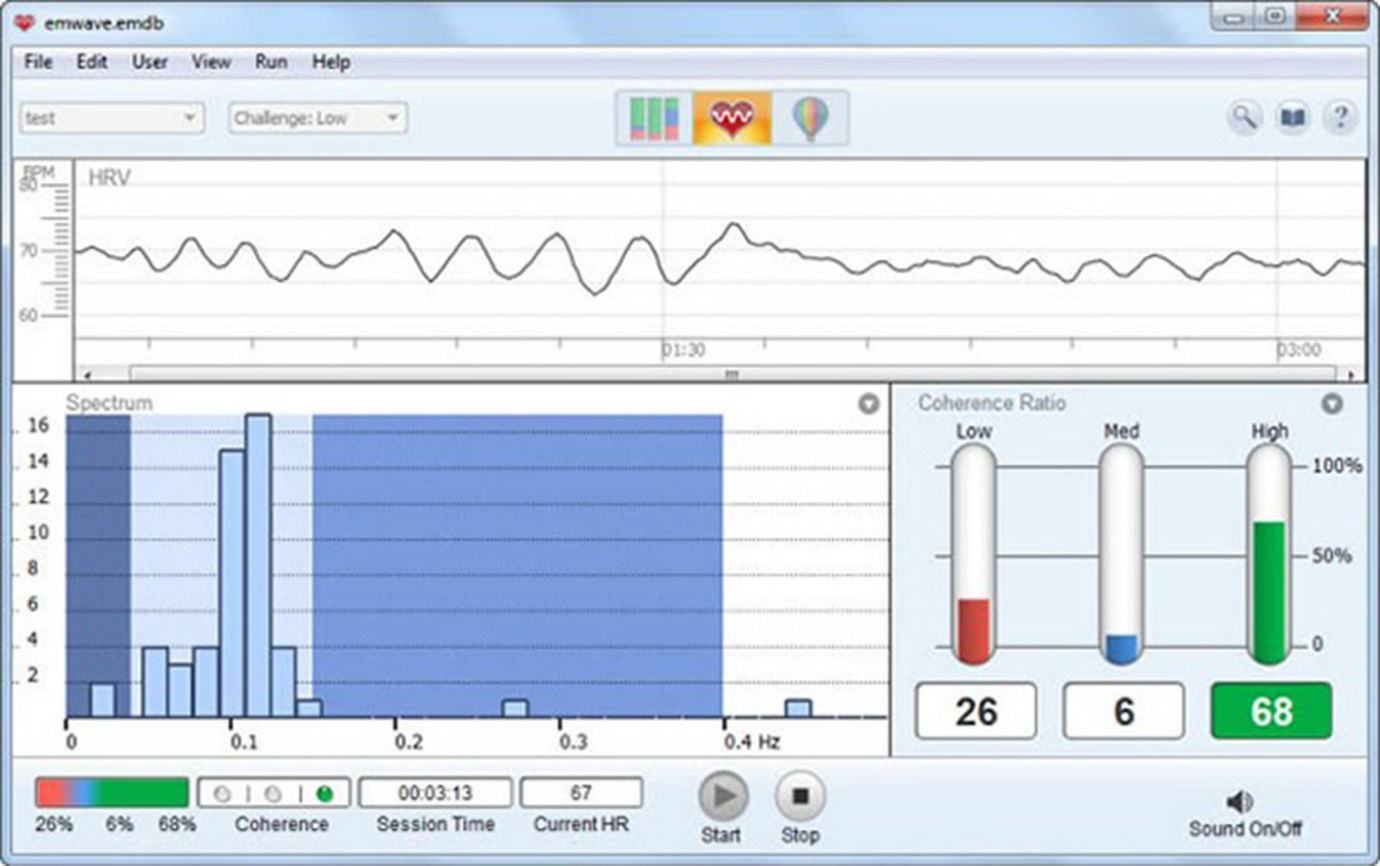
**Display of short-term HRV activity**. Credit: Institute of HeartMath.

The interactions between autonomic neural activity, BP, and respiratory control systems produce short-term rhythms in HRV measurements (Hirsch and Bishop, 1981, 1996; McCraty et al., 2009) (Figure 6). The most common form for observing these changes is the heart rate tachogram, a plot of a sequence of time intervals between R waves. Efferent sympathetic and parasympathetic activity is integrated in and with the activity occurring in the heart's intrinsic nervous system, including the afferent signals occurring from the mechanosensitive and chemosensory neurons, all of which contribute to beat-to-beat changes. HRV is thus considered a measure of neurocardiac function that reflects heart–brain interactions and ANS dynamics.

Circadian rhythms, core body temperature, metabolism, hormones, and intrinsic rhythms generated by the heart all contribute to lower frequency rhythms [e.g., very-low-frequency (VLF) and ultra-low-frequency (ULF)] that extend below 0.04 Hz. Due to their long time periods, researchers use 24-h HRV recordings to provide comprehensive assessment of their fluctuations (Kleiger et al., 2005). In concert, these multiple influences create a dynamic physiological control system that is never truly at rest and is certainly never static. In healthy individuals, it remains responsive and resilient, primed and ready to react when needed.

## How is HRV detected?

Clinicians use ECG or photoplethysmograph (PPG) sensors to detect the interbeat interval (IBI). While the ECG method had been considered to be more accurate than the PPG method because early software algorithms could more easily detect the sharp upward spike of the R wave than the curved peak of the blood volume pulse signal, newer algorithms have improved peak detection from the pulse wave. The ECG method should be used when recordings are contaminated by frequent abnormal beats (e.g., premature ventricular contractions), since the ECG's morphology and timing properties allow software algorithms to discriminate normal sinus beats from ectopic beats (Mateo et al., 2011).

All HRV assessments are calculated from an IBI file. However, in some cases there can be differences in the IBI files derived from ECG and PPG data. Several studies have shown that when the recordings are taken during a resting state (sitting quietly as done in most resting baseline recordings), the IBI values between ECG and PPG are highly correlated (Giardino et al., 2002; Schafer and Vagedes, 2013). However, during ambulatory monitoring or when a person experiences a stressor strong enough to activate the sympathetic system, there can be significant differences due to changes in pulse transit time (the time it takes the BP wave to propagate from the heart to the periphery), which result from changes in the elasticity of the arteries. When arteries stiffen due to sympathetic activation, the BP wave travels faster. The accuracy of HRV measurements is primarily determined by the sampling rate of the data acquisition system. Kuusela (2013) recommends a sampling rate of 200 Hz unless overall variability among RR intervals is unusually low, as in case of heart failure. In contrast, Berntson et al. (2007) recommend a minimum sampling rate of 500–1000 Hz. However, for many applications, like HRV biofeedback (HRVB), a sampling rate of 126 Hz may be adequate.

**Figure 7:**
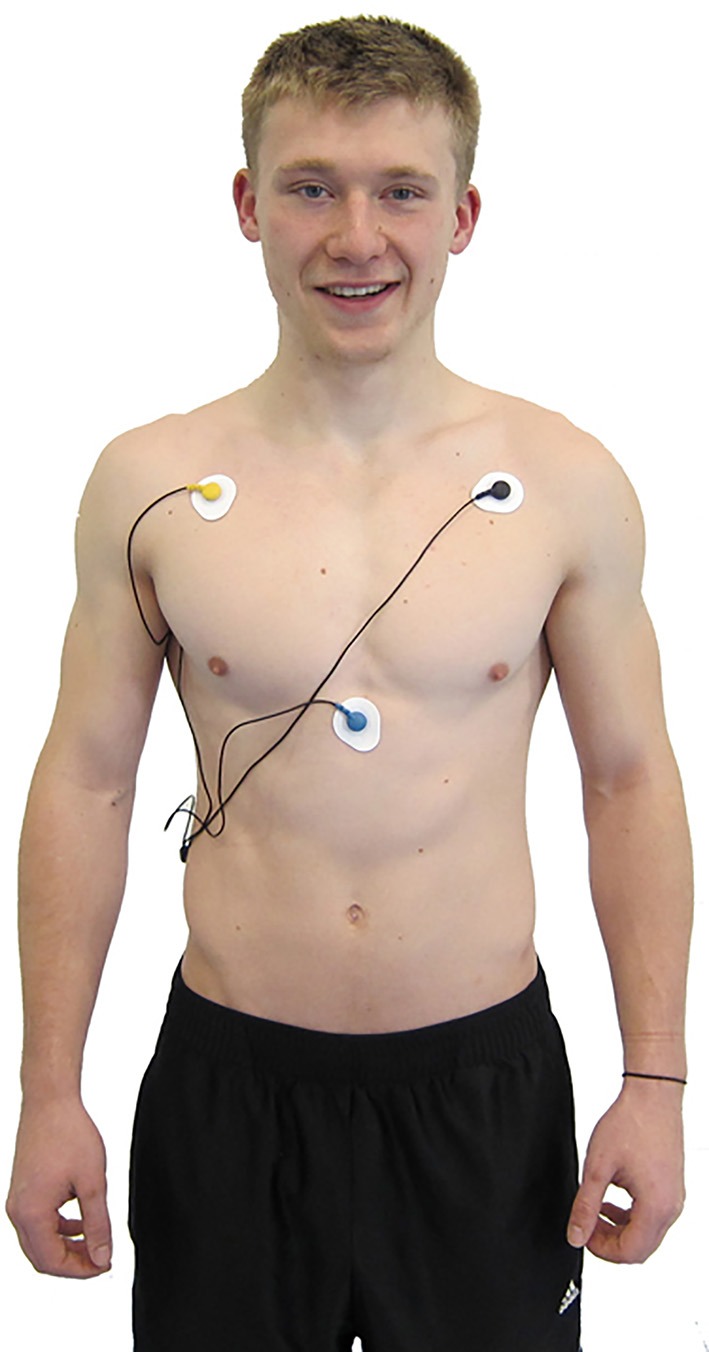
**ECG electrode placement**. Credit: Truman State University Center for Applied Psychophysiology.

There are many ECG configurations, with varying numbers of leads, used for ambulatory and stationary monitoring. For example, a standard three-lead ECG chest placement locates active and reference electrodes over the right and left coracoid processes, respectively, and a second active electrode over the xiphoid process (Figure 7).

## Why is HRV important?

An optimal level of variability within an organism's key regulatory systems is critical to the inherent flexibility and adaptability or resilience that epitomizes healthy function and well-being. While too much instability is detrimental to efficient physiological functioning and energy utilization, too little variation indicates depletion or pathology.

### HRV is a marker for disease and adaptability

The clinical importance of HRV was noted as far back as 1965 when it was found that fetal distress is preceded by alterations in HRV before any changes occur in heart rate itself (Hon and Lee, 1963). In the 1970s, HRV analysis was shown to predict autonomic neuropathy in diabetic patients before the onset of symptoms (Ewing et al., 1976). Low HRV has since been confirmed as a strong, independent predictor of future health problems and as a correlate of all-cause mortality (Tsuji et al., 1994; Dekker et al., 1997). Reduced HRV is also observed in patients with autonomic dysfunction, including anxiety, depression, asthma, and sudden infant death (Kazuma et al., 1997; Carney et al., 2001; Agelink et al., 2002; Giardino et al., 2004; Lehrer et al., 2004; Cohen and Benjamin, 2006).

Based on indirect evidence, reduced HRV may correlate with disease and mortality because it reflects reduced regulatory capacity, which is the ability to adaptively respond to challenges like exercise or stressors. For example, patients with low overall HRV demonstrated reduced cardiac regulatory capacity and an increased likelihood of prior myocardial infarction (MI). In this sample, a measure of cardiac autonomic balance did not predict previous MIs (Berntson et al., 2008).

Patient age may mediate the relationship between reduced HRV and regulatory capacity. HRV declines with age (Umetani et al., 1998) and aging often involves nervous system changes, like loss of neurons in the brain and spinal cord, which may degrade signal transmission (Jäncke et al., 2014) and reduce regulatory capacity.

Reduced regulatory capacity may contribute to functional gastrointestinal disorders, inflammation, and hypertension. While patients with functional gastrointestinal disorders often have reduced HRV (Gevirtz, 2013), HRVB has increased vagal tone and improved symptom ratings in these patients (Sowder et al., 2010).

The PNS may help regulate inflammatory responses via a cholinergic anti-inflammatory system (Tracey, 2007). While the experimental administration of lipopolysaccharide to healthy volunteers decreases HRV and vagal tone (Jan et al., 2009), HRVB training has reduced the symptoms produced by this intervention (Lehrer et al., 2010).

Hypertensive patients often present with reduced baroreflexes and HRV (Schroeder et al., 2003). HRVB can increase baroreflex gain, which is the amplitude of HR changes, and HRV, and decrease BP (Lehrer, 2013). Several randomized-controlled studies have documented BP reductions in hypertensive patients who received HRVB (Elliot et al., 2004; Reineke, 2008).

HRV is also an indicator of psychological resiliency and behavioral flexibility, reflecting the individual's capacity to adapt effectively to changing social or environmental demands (Beauchaine, 2001; Berntson et al., 2008). More recently, several studies have shown an association between higher levels of resting HRV and performance on cognitive performance tasks requiring the use of executive functions (Thayer et al., 2009) and that HRV, especially HRV-coherence, can be increased in order to produce improvements in cognitive function as well as a wide range of clinical outcomes, including reduced health care costs (Lehrer et al., 2003, 2008; McCraty et al., 2003; Bedell and Kaszkin-Bettag, 2010; Alabdulgader, 2012).

### HRV analysis methods

It was recognized as far back as 1979 that nomenclature, analytical methods, and definitions of HRV measures required standardization. Therefore, an International Task Force consisting of members from the European Society of Cardiology and the North American Society for Pacing and Electrophysiology was established. Their report was published in Task Force (1996).

HRV can be assessed with various analytical approaches, although the most commonly used are frequency domain or power spectral density (PSD) analysis and time domain analysis. In both methods, the time intervals between each successive normal QRS complex are first determined. All abnormal beats not generated by sinus node depolarizations are eliminated from the record.

**Figure 8:**
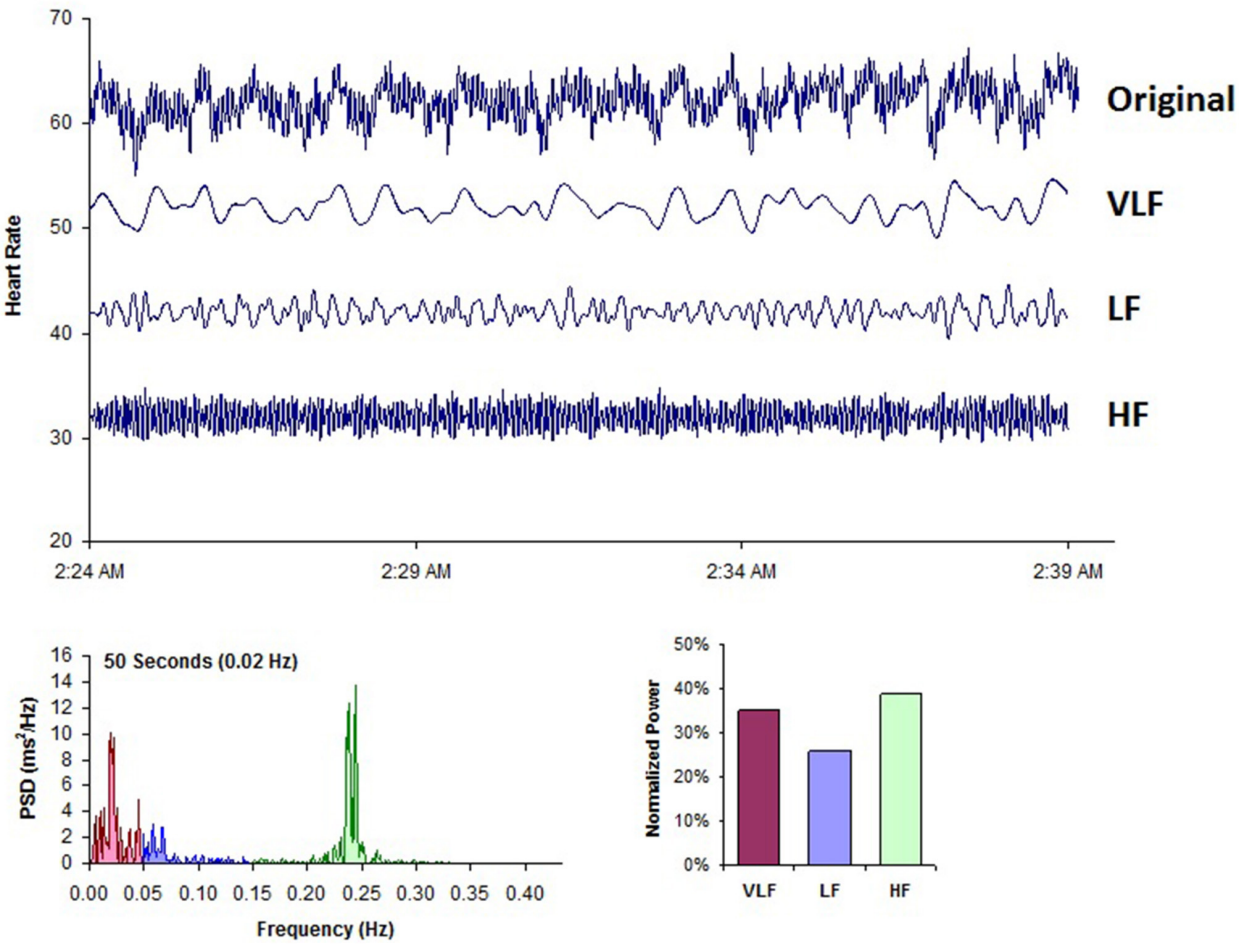
**This figure shows a typical HRV recording over a 15-min period during resting conditions in a healthy individual**. The top trace shows the original HRV waveform. Filtering techniques were used to separate the original waveform into VLF, LF, and HF bands as shown in the lower traces. The bottom of the figure shows the power spectra (left) and the percentage of power (right) in each band. Credit: Institute of HeartMath.

Analogous to the EEG, we can use power spectral analysis to separate HRV into its component rhythms that operate within different frequency ranges (Figure 8). PSD analysis provides information of how power is distributed (the variance and amplitude of a given rhythm) as a function of frequency (the time period of a given rhythm). The main advantages of spectral analysis over the time domain measures are that it supplies both frequency and amplitude information about the specific rhythms that exist in the HRV waveform, providing a means to quantify the various oscillations over any given period in the HRV recording. The values are expressed as the PSD, which is the area under the curve (peak) in a given segment of the spectrum. The power or height of the peak at any given frequency indicates the amplitude and stability of the rhythm. The frequency reflects the period of time over which the rhythm occurs. For example, a 0.1 Hz frequency has a period of 10 s. In order to understand how power spectral analysis distinguishes the various underlying physiological mechanisms that are reflected in the heart's rhythm, a brief review of these underlying physiological mechanisms follows.

Figure 8 shows a typical example of an HRV recoding from an adult human at rest. Using filtering techniques, the high-frequency (HF), low-frequency (LF), and VLF bands have been extracted from the original HRV signal and spectral power has been calculated for each band.

## Sources of HRV

The Task Force report (1996) divided heart rhythm oscillations into four primary frequency bands. These included the HF, LF, VLF, and ULF bands. The Task Force report also stated that the analysis should be done on 5-min segments, although other recording periods are often used. When other recording lengths are analyzed and reported, the length of the recording should be reported since this has large effects on both HRV frequency and time domain values.

### High-frequency band

The HF spectrum is the power in each of the 288 5-min segments (monitored during a 24-h period) in the range from 0.15 to 0.4 Hz. This band reflects parasympathetic or vagal activity and is frequently called the respiratory band because it corresponds to the HR variations related to the respiratory cycle. These HR changes are known as respiratory sinus arrhythmia (RSA). Heart rate accelerates during inspiration and slows during expiration. During inhalation, the cardiovascular center inhibits vagal outflow resulting in speeding the heart rate. Conversely, during exhalation, it restores vagal outflow resulting in slowing the heart rate via the release of acetylcholine (Eckberg and Eckberg, 1982). The magnitude of the oscillation is variable, but can usually be exaggerated by slow, deep breathing.

The modulation of vagal tone helps maintain the dynamic autonomic regulation important for cardiovascular health. Deficient vagal inhibition is implicated in increased morbidity (Thayer et al., 2010). The mechanism linking the variability of HR to respiration is complex and involves both central and reflex interactions. A large number of studies have shown that total vagal blockade essentially eliminates HF oscillations and reduces the power in the LF range (Pomeranz et al., 1985; Malliani et al., 1991).

Reduced parasympathetic (high frequency) activity has been found in a number of cardiac pathologies and in patients under stress or suffering from panic, anxiety, or worry. Lowered parasympathetic activity may primarily account for reduced HRV in aging (Umetani et al., 1998). In younger healthy individuals, it is not uncommon to see an obvious increase in the HF band at night with a decrease during the day (Lombardi et al., 1996; Otsuka et al., 1997).

### Low-frequency band

**Figure 9:**
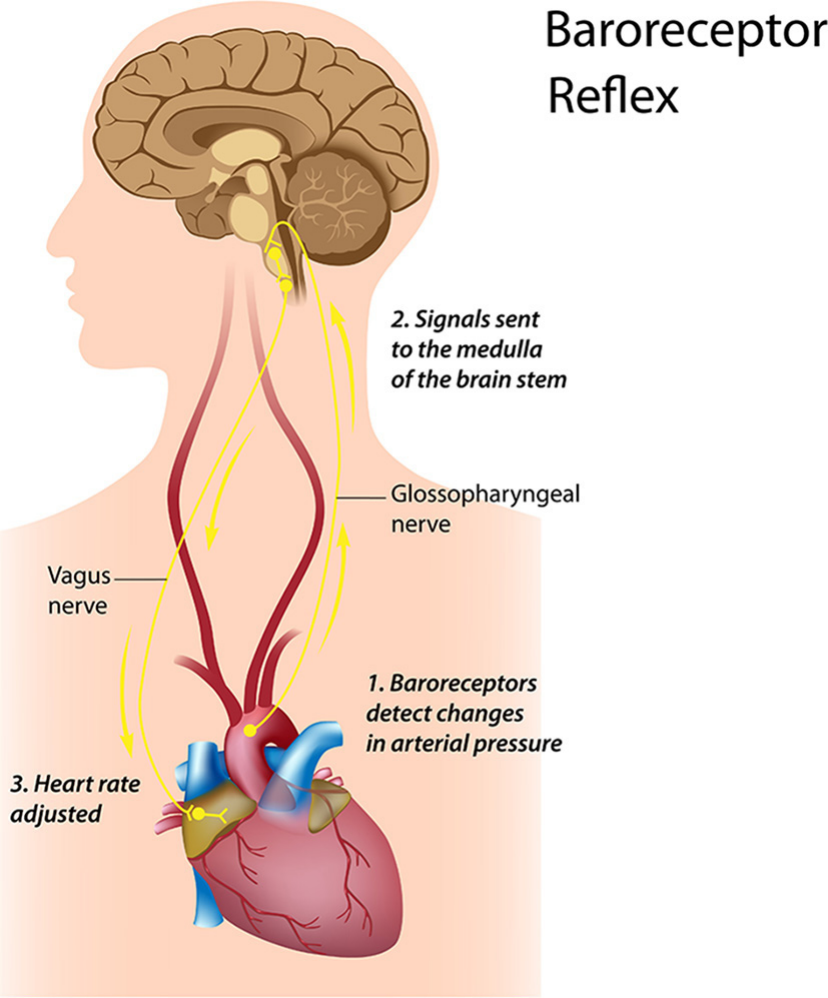
**Credit: Alila Sao Mai/Shutterstock.com**.

The LF band ranges between 0.04 and 0.15 Hz. This region was previously called the “baroreceptor range” or “mid-frequency band” by many researchers, since it primarily reflects baroreceptor activity while at rest (Malliani, 1995). The vagus nerves are a major conduit though which afferent neurological signals from the heart and other visceral organs are relayed to the brain, including the baroreflex signals (De Lartique, 2014). Baroreceptors are stretch-sensitive mechanoreceptors located in the chambers of the heart and vena cavae, carotid sinuses (which contain the most sensitive mechanoreceptors), and the aortic arch (Figure 9). When BP rises, the carotid and aortic tissues are distended, resulting in increased stretch and, therefore, increased baroreceptor activation. At normal resting BPs, many baroreceptors actively report BP information and the baroreflex modulates autonomic activity.

Active baroreceptors generate action potentials (“spikes”) more frequently. The greater their stretch or detection of an increased rate of change, the more frequently baroreceptors fire action potentials. Baroreceptor action potentials are relayed to the NST in the medulla, which uses baroreceptor firing frequency to measure BP. Increased activation of the NST inhibits the vasomotor center and stimulates the vagal nuclei. The end-result of baroreceptor activations tuned to pressure increases is inhibition of the SNS and activation of the PNS. By coupling sympathetic inhibition with parasympathetic activation, the baroreflex maximizes BP reduction when BP is detected as too high. Sympathetic inhibition reduces peripheral resistance, while parasympathetic activation depresses HR (reflex bradycardia) and contractility. In a similar manner, sympathetic activation, along with inhibition of vagal outflow, allows the baroreflex to elevate BP. Baroreflex gain is commonly calculated as the beat-to-beat change in HR per unit of change in BP. Decreased baroreflex gain is related to impaired regulatory capacity and aging.

The existence of a cardiovascular system resonance frequency, which is caused by the delay in the feedback loops in the baroreflex system, has been long established (Vaschillo et al., 2011). Lehrer et al. have proposed that each individual's cardiovascular system has a unique resonance frequency, which can be identified by measuring HRV while an individual breathes between 7.5 and 4.5 breaths per minute (Lehrer et al., 2013). When the cardiovascular system oscillates at this frequency, there is a distinctive high-amplitude peak in the HRV power spectrum around 0.1 Hz. Most mathematical models show that the resonance frequency of the human cardiovascular system is determined by the feedback loops between the heart and brain (deBoer et al., 1987; Baselli et al., 1994). In humans and many other mammals, the resonance frequency of the system is approximately 0.1 Hz, which is equivalent to a 10-s rhythm.

The sympathetic system does not appear to produce rhythms much above 0.1 Hz, while the parasympathetic system can be observed to affect heart rhythms down to 0.05 Hz (20-s rhythm). During periods of slow respiration rates, vagal activity can easily generate oscillations in the heart rhythms that cross over into the LF band (Ahmed et al., 1982; Tiller et al., 1996; Lehrer et al., 2003). Therefore, respiratory-related efferent vagally-mediated influences are particularly present in the LF band when respiration rates are below 8.5 breaths per minute or 7-s periods (Brown et al., 1993; Tiller et al., 1996) or when an individual sighs or takes a deep breath.

In ambulatory 24-h HRV recordings, it has been suggested that the LF band also reflects sympathetic activity and the LF/HF ratio has been controversially reported as an assessment of the balance between sympathetic and parasympathetic activity (Pagani et al., 1984, 1986). A number of researchers (Tiller et al., 1996; Eckberg, 1997; Porges, 2007; Rahman et al., 2011; Heathers, 2012) have challenged this perspective and have persuasively argued that in resting conditions, the LF band reflects baroreflex activity and not cardiac sympathetic innervation.

The perspective that the LF band reflects sympathetic activity came from observations of 24-h ambulatory recordings where there are frequent sympathetic activations primarily due to physical activity, but also due to emotional stress reactions, which can create oscillations in the heart rhythms that cross over into the lower part of the LF band. In long-term ambulatory recordings, the LF band fairly approximates sympathetic activity when increased sympathetic activity occurs (Axelrod et al., 1987). This will be discussed in more detail in the VLF section. Unfortunately, some authors have assumed that this interpretation was also true of short-term resting recordings and have confused slower breathing-related increases in LF power with sympathetic activity, when in reality it is almost entirely vagally mediated. Remember that the baroreflex is primarily vagally mediated (Keyl et al., 1985).

Porges (2007) suggests that under conditions when participants pace their breathing at 0.1 Hz (10-s rhythm or 6 breaths per minute), which is a component of many HRVB training protocols, the LF band includes the summed influence of both efferent vagal pathways (myelinated and unmyelinated, which reflects total cardiac vagal tone).

### Autonomic balance and the LF/HF ratio

The autonomic balance hypothesis assumes that the SNS and PNS competitively regulate SA node firing, where increased SNS activity is paired with decreased PNS activity. While some orthostatic challenges can produce reciprocal changes in SNS activation and vagal withdrawal, psychological stressors can also result in independent changes in SNS or PNS activity. It is now generally accepted that both branches of the ANS can be simultaneously active (Berntson and Cacioppo, 1999). Therefore, the relationship between the SNS and PNS in generating LF power appears to be complex, non-linear, and dependent upon the experimental manipulation employed (Berntson et al., 1997; Billman, 2013).

The ratio of LF to HF power is called the LF/HF ratio. The interpretation of the LF/HF ratio is controversial due to the issues regarding the LF band described above. However, once the mechanisms are understood as well as the importance of the recording context (i.e., ambulatory vs. resting conditions and normal vs. paced breathing), the controversy is resolved. Recall that the power in the LF band can be influenced by vagal, sympathetic, and baroreflex mechanisms depending on the context, whereas HF power is produced by the efferent vagal activity due to respiratory activity. It is often assumed that a low LF/HF ratio reflects greater parasympathetic activity relative to sympathetic activity due to energy conservation and engaging in “tend-and-befriend” behaviors (Taylor, 2006). However, this ratio is often shifted due to reductions in LF power. Therefore, the LF/HR ratio should be interpreted with caution and the mean values of HF and LF power taken into consideration. In contrast, a high LF/HF ratio may indicate higher sympathetic activity relative to parasympathetic activity as can be observed when people engage in meeting a challenge that requires effort and increased SNS activation. Again, the same cautions must be taken into consideration, especially in short-term recordings.

### Very-low-frequency band

The VLF band is the power in the HRV power spectrum range between 0.0033 and 0.04 Hz. Although all 24-h clinical measures of HRV reflecting low HRV are linked with increased risk of adverse outcomes, the VLF band has stronger associations with all-cause mortality than the LF and HF bands (Tsuji et al., 1994, 1996; Hadase et al., 2004; Schmidt et al., 2005). Low VLF power has been shown to be associated with arrhythmic death (Bigger et al., 1992) and PTSD (Shah et al., 2013). Additionally, low power in this band has been associated with high inflammation in a number of studies (Carney et al., 2007; Lampert et al., 2008) and has been correlated with low levels of testosterone, while other biochemical markers, such as those mediated by the HPA axis (e.g., cortisol), did not (Theorell et al., 2007).

Historically, the physiological explanation and mechanisms involved in the generation of the VLF component have not been as well defined as the LF and HF components, and this region has been largely ignored. Long-term regulation mechanisms and ANS activity related to thermoregulation, the renin-angiotensin system, and other hormonal factors may contribute to this band (Akselrod et al., 1981; Cerutti et al., 1995; Claydon and Krassioukov, 2008). Recent work by Dr. Andrew Armour has shed new light on the mechanisms underlying the VLF rhythm and suggests that we may have to reconsider both the mechanisms and importance of this band.

**Figure 10:**
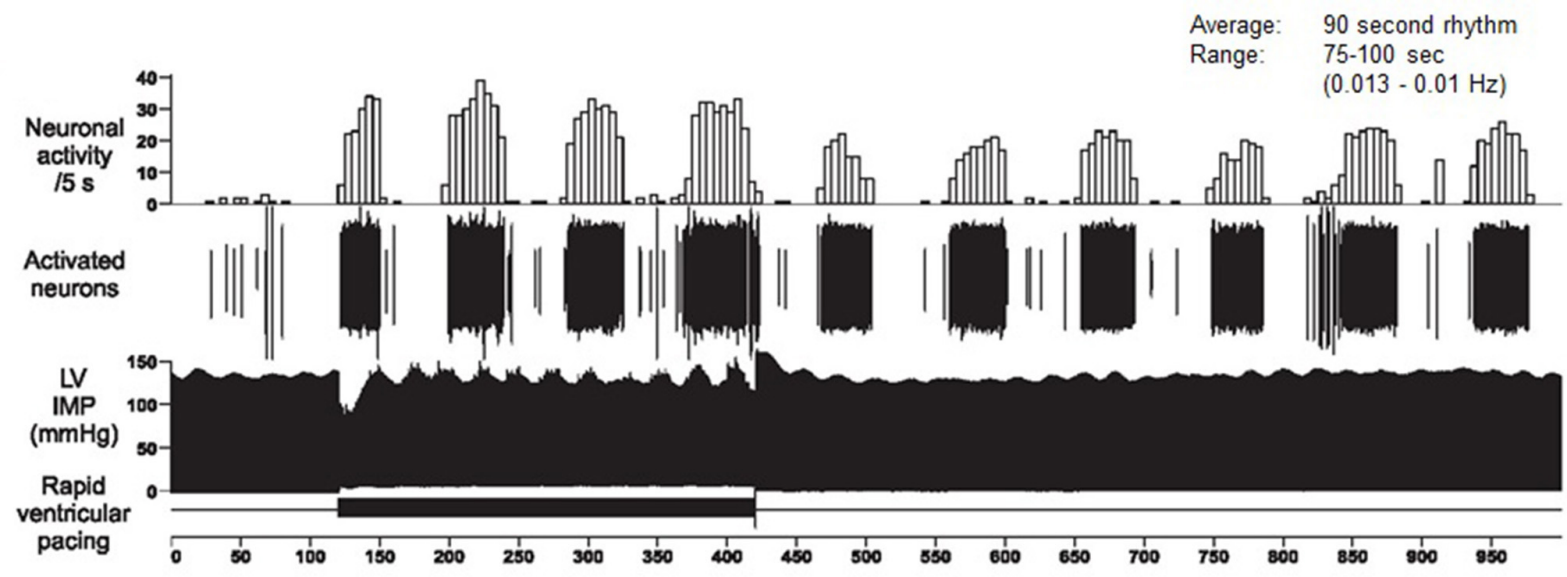
**Long-term single-neuron recordings from an afferent neuron in the intrinsic cardiac nervous system in a beating dog heart**. The top row shows neural activity, the second row, the actual neural recording, and the third row, the left ventricular pressure. This intrinsic rhythm has an average period of 90 s with a range between 75 and 100 s (0.013–0.01 Hz), which falls within the VLF band. Credit: Dr. Andrew Armour and the Institute of HeartMath.

Dr. Armour's group has developed the technology to obtain long-term single-neuron recordings from a beating heart, and simultaneously, from extrinsic cardiac neurons (Armour, 2003). Figure 10 shows the VLF rhythm obtained from an afferent neuron located in the intrinsic cardiac nervous system in a dog heart. In this case, the VLF rhythm is generated from intrinsic sources and cannot be explained by sources such as movement. The black area in the bottom of the figure labeled “rapid ventricular pacing” shows the time period where efferent spinal neurons were stimulated. The resulting increase in efferent sympathetic activity (bottom row) clearly elevates the amplitude of the single afferent neuron's intrinsic VLF rhythm (top row).

This work, combined with findings by Kember et al. (2000, 2001), implies that the VLF rhythm is generated by the stimulation of afferent sensory neurons in the heart, which in turn activate various levels of the feedback and feed-forward loops in the heart's intrinsic cardiac nervous system, as well as between the heart, the extrinsic cardiac ganglia, and spinal column. Thus, the VLF rhythm is produced by the heart itself and is an intrinsic rhythm that appears to be fundamental to health and well-being. Dr. Armour has observed that when the amplitude of the VLF rhythm at the neural level is diminished, an animal subject is in danger and will expire shortly if they proceed with the research procedures (personal communication with McCraty). Sympathetic blockade does not affect VLF power and VLF activity is seen in tetraplegics, whose SNS innervation of the heart and lungs is disrupted (Task Force, 1996; Berntson et al., 1997). These findings further support a cardiac origin of the VLF rhythm.

In healthy individuals, there is an increase in VLF power that occurs during the night and peaks before waking (Huikuri et al., 1994; Singh et al., 2003). This increase in autonomic activity may correlate with the morning cortisol peak.

In summary, experimental evidence suggests that the VLF rhythm is intrinsically generated by the heart and that the amplitude and frequency of these oscillations are modulated by efferent sympathetic activity. Normal VLF power appears to indicate healthy function, and increases in resting VLF power may reflect increased sympathetic activity. The modulation of the frequency of this rhythm due to physical activity (Bernardi et al., 1996), stress responses, and other factors that increase efferent sympathetic activation can cause it to cross over into the lower region of the LF band during ambulatory monitoring or during short-term recordings when there is a significant stressor.

### Ultra-low-frequency band

The ULF band falls below 0.0033 Hz (333 s or 5.6 min). Oscillations or events in the heart rhythm with a period of 5 min or greater are reflected in this band and it can only be assessed with 24-h and longer recordings (Kleiger et al., 2005). The circadian oscillation in heart rate is the primary source of the ULF power, although other very slow-acting regulatory processes, such as core body temperature regulation, metabolism, and the renin-angiotensin system likely add to the power in this band (Bonaduce et al., 1994; Task Force, 1996). Different psychiatric disorders show distinct circadian patterns in 24-h heart rates, particularly during sleep (Stampfer, 1998; Stampfer and Dimmitt, 2013).

**Figure 11:**
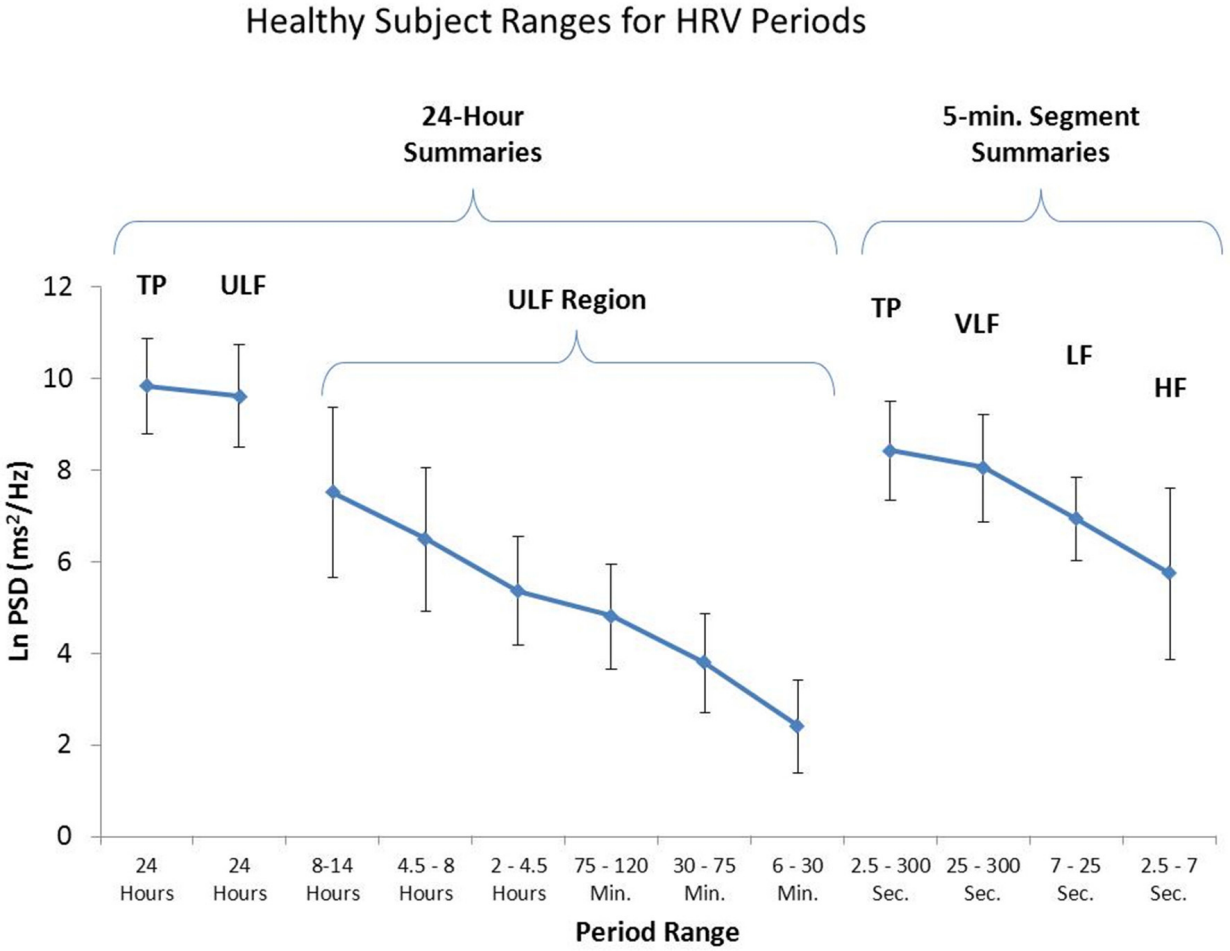
**This figure shows the power in the various frequency bands for 24-h HRV and 95% confidence intervals for each of the bands**. The left side of the figure reveals a number of slower rhythms that make up the ULF band. The analysis was conducted using the healthy sample described in Umetani et al. (1998). The right side of the figure shows an analysis of the same data performed on 5-min segments as is traditionally done. Credit: Institute of HeartMath.

The Task Force report (1996) stated that analysis of 24-h recordings should divide the record into 5-min segments and that HRV analysis should be performed on the individual segments prior to the calculation of mean values. This effectively filters out any oscillations with periods longer than 5 min. However, as shown in Figure 11, when spectral analysis is applied to entire 24-h records, several lower frequency rhythms are easily detected in healthy individuals. At the present time, the clinical relevance of these lower frequency rhythms is unknown, largely due to the Task Force guidelines that eliminate their presence from most analysis procedures.

## Time domain measurements of HRV

Time domain measures are the simplest to calculate and include the mean normal-to-normal (NN) intervals during the entire recording and other statistical measures such as the standard deviation between NN intervals (SDNN). However, time domain measures do not provide a means to adequately quantify autonomic dynamics or determine the rhythmic or oscillatory activity generated by the different physiological control systems. Since they are always calculated the same way, data collected by different researchers are comparable, but only if the recording lengths are exactly the same and the data are collected under the same conditions.

Time domain indices quantify the amount of variance in the IBI using statistical measures. For 24-h recordings, the three most important time domain measures are the SDNN, the SDNN index, and the RMSSD. For short-term assessments, the SDNN, RMSSD, pNN50, and HR Max – HR Min are most commonly reported.

### SDNN

The SDNN is the standard deviation of the normal (NN) sinus-initiated IBI measured in milliseconds. This measure reflects the ebb and flow of all the factors that contribute to heart rate variability (HRV). In 24-h recordings, the SDNN is highly correlated with ULF and total power (Umetani et al., 1998). In short-term resting recordings, the primary source of the variation is parasympathetically-mediated RSA, especially with slow, paced breathing protocols.

SDNN values are highly correlated with the lower frequency rhythms discussed earlier (Table 1). Low age-adjusted values predict both morbidity and mortality. Classification within a higher SDNN category is associated with a higher probability of survival. For example, patients with moderate SDNN values, 50–100 ms, have a 400% lower risk of mortality than those with low values, 0–50 ms, in 24-h recordings (Kleiger et al., 1987).

**Table 1:** **Correlations between time and frequency domain measures in 24-h recordings**.

	**HR (ms)**	**N-D delta**	**SDNN**	**Ln total power**	**Ln ULF**	**SDANN**	**SDNN index**	**Ln 5-min total power**	**Ln 5-min VLF**	**Ln 5-min LF**	**Ln 5-min HF**	**Ln RMSSD**	**Ln LF/HF**
HR (ms)	1												
N-D delta	0.29	1											
SDNN	0.61	0.66	1										
Ln total power	0.55	0.66	0.98	1									
Ln ULF	0.47	0.67	0.95	0.99	1								
SDANN	0.47	0.70	0.96	0.97	0.98	1							
SDNN index	0.72	0.43	0.79	0.73	0.62	0.62	1						
Ln 5-min total power	0.71	0.40	0.78	0.71	0.60	0.61	0.99	1					
Ln 5-min VLF	0.74	0.49	0.83	0.80	0.70	0.68	0.96	0.93	1				
Ln 5-min LF	0.57	0.27	0.63	0.61	0.49	0.48	0.87	0.84	0.81	1			
Ln 5-min HF	0.36	0.38	0.56	0.54	0.44	0.44	0.79	0.75	0.68	0.75	1		
Ln RMSSD	0.54	0.41	0.68	0.64	0.54	0.54	0.90	0.86	0.80	0.82	0.95	1	
Ln LF/HF	−0.02	−0.31	−0.27	−0.24	−0.20	−0.21	−0.37	−0.34	−0.27	−0.20	−0.80	−0.66	−0.20

*Credit: Institute Of Heartmath*.

### SDANN

The SDANN is the standard deviation of the average NN intervals (mean heart rate) for each of the 5-min segments during a 24-h recording. Like the SDNN, it is measured and reported in milliseconds. This index is correlated with the SDNN and is generally considered redundant.

### SDNN index

The SDNN index is the mean of the standard deviations of all the NN intervals for each 5-min segment of a 24-h HRV recording. Therefore, this measurement only estimates variability due to the factors affecting HRV within a 5-min period. It is calculated by first dividing the 24-h record into 288 5-min segments and then calculating the standard deviation of all NN intervals contained within each segment. The SDNN Index is the average of these 288 values. The SDNN index is believed to primarily measure autonomic influence on HRV. This measure tends to correlate with VLF power over a 24-h period.

### RMSSD

The RMSSD is the root mean square of successive differences between normal heartbeats. This value is obtained by first calculating each successive time difference between heartbeats in milliseconds. Then, each of the values is squared and the result is averaged before the square root of the total is obtained. The RMSSD reflects the beat-to-beat variance in heart rate and is the primary time domain measure used to estimate the vagally-mediated changes reflected in HRV. While the RMSSD is correlated with HF power (Kleiger et al., 2005), the influence of respiration rate on this index is uncertain (Schipke et al., 1999; Pentillä et al., 2001). Lower RMSSD values are correlated with higher scores on a risk inventory of sudden unexplained death in epilepsy (DeGiorgio et al., 2010).

### pNN50

The pNN50 is the percentage of adjacent NN intervals that differ from each other by more than 50 ms. It is correlated with the RMSSD and HF power. However, the RMSSD typically provides a better assessment of RSA (especially in older subjects) and most researchers prefer it to the pNN50 (Otzenberger et al., 1998).

### HR max – HR min

HR Max – HR Min is the average difference between the highest and lowest HRs during each respiratory cycle. This measure is especially used for assessment in paced breathing protocols and is highly correlated with the SDNN and RMSSD.

## Polyvagal theory

As previously discussed, increased efferent activity in the vagal nerves (also called the 10th cranial nerve) slows the heart rate, yet has an opposite effect in the lungs as it increases bronchial tone. According to Porges' (2011) polyvagal theory, the ANS must be considered a “system,” with the vagal nerves containing specialized subsystems that regulate competing adaptive responses. His theory proposes competing roles for the unmyelinated fibers in the vagus, which originate in the dorsal motor complex, and newer myelinated nerves, which originate in the nucleus ambiguus. He hypothesizes that the unmyelinated fibers are involved in regulating the “freeze response” and respond to threats through immobilization, feigning death, passive avoidance, and shutdown (the freeze response).

In Porges' view, the evolution of the ANS was central to the development of emotional experience and affective processes central to social behavior. As human beings, we are not limited to fight, flight, or freezing behavioral responses. We can self-regulate and initiate pro-social behaviors (e.g., the tend-and-befriend response) when we encounter stressors. Porges calls this the social engagement system and the theory suggests that this system depends upon the healthy functioning of the myelinated vagus, a vagal brake, which allows for self-regulation and ability to calm ourselves and inhibit sympathetic outflow to the heart. This implies that standardized assessment of vagal tone could serve as a potential marker for one's ability to self-regulate.

The theory suggests that the evolution and healthy function of the ANS sets the limits or boundaries for the range of one's emotional expression, quality of communication, and ability to self-regulate emotions and behaviors. The theory describes the details of the anatomical connections from higher brain structures with the centers involved in autonomic regulation and argues that the afferent systems are an important aspect of the ANS. The theory provides insights into the adaptive nature of physiological states and suggests these states support different types or classes of behavior (Porges, 2011).

The SNS, in concert with the endocrine system, responds to threats to our safety through mobilization, fight-or-flight, and active avoidance. The SNS responds more slowly and for a longer period of time (i.e., more than a few seconds) than the vagus system. According to this theory, quality communication and pro-social behaviors can only be effectively engaged when these defensive circuits are inhibited.

## Neurovisceral integration: the central autonomic network model

Thayer and Lane (2000) outline a neurovisceral integration model that describes how a set of neural structures involved in cognitive, affective, and autonomic regulation are related to HRV and cognitive performance. In this complex systems model, the anatomical details of a central autonomic network (CAN) are described that link the NST in the brainstem with forebrain structures (including the anterior cingulate, insula, ventromedial prefrontal cortex, amygdala, and hypothalamus) through feedback and feed-forward loops. They propose that this network is an integrated system for internal system regulation by which the brain controls visceromotor, neuroendocrine, and behavioral responses that are critical for goal-directed behavior, adaptability, and health.

Thayer et al. (2012) contend that dynamic connections between the amygdala and medial prefrontal cortex, which evaluate threat and safety, help regulate HRV through their connections with the NST. They propose that vagally-mediated HRV is linked to higher-level executive functions and that HRV reflects the functional capacity of the brain structures that support working memory and emotional and physiological self-regulation. They hypothesize that vagally-mediated HRV is positively correlated with prefrontal cortical performance and the ability to inhibit unwanted memories and intrusive thoughts. In their model, when the CAN decreases prefrontal cortical activation, HR increases and HRV decreases. The prefrontal cortex can be taken “offline” when individuals perceive that they are threatened. Prolonged prefrontal cortical inactivity can lead to hypervigilance, defensiveness, and social isolation (Thayer et al., 2009).

The CAN model predicts reduced HRV and vagal activity in anxiety. Friedman (2007) argues that anxiety is associated with abnormal ANS cardiac control. HRV indices consistently show low vagal activity in patients diagnosed with anxiety disorders. This finding challenges the completeness of the sympathetic overactivation explanation of anxiety. Friedman observes that “metaphorically, investigators were searching for a ‘sticky accelerator’ while overlooking the possibility of ‘bad brakes’” (p. 186). From his perspective, anxiety disorders can involve varying degrees of sympathetic overactivation and parasympathetic underactivation.

## The psychophysiological coherence model

McCraty and Childre (2010) at the Institute of HeartMath also take a dynamic systems approach that focuses on increasing individuals' self-regulatory capacity by inducing a physiological shift that is reflected in the heart's rhythms. They theorize that rhythmic activity in living systems reflects the regulation of interconnected biological, social, and environmental networks. The coherence model also suggests that information is encoded in the dynamic patterns of physiological activity. For example, information is encoded in the time interval between action potentials and patterns in the pulsatile release of hormones. They suggest that the time intervals between heartbeats (HRV) also encode information which is communicated across multiple systems, which helps synchronize the system as whole. The afferent pathways from the heart and cardiovascular system are given more relevance in this model due the significant degree of afferent cardiovascular input to the brain and the consistent generation of dynamic patterns generated by the heart. It is their thesis that positive emotion in general, as well as self-induced positive emotions, shift the system as a whole into a more globally coherent and harmonious physiological mode associated with improved system performance, ability to self-regulate, and overall well-being.

They use the term “physiological coherence” to describe the orderly and stable rhythms generated by living systems. Physiological coherence is used broadly and includes all of the specific approaches for quantifying the various types of coherence measures, such as cross-coherence (frequency entrainment between respiration, BP, and heart rhythms), or synchronization among systems (e.g., synchronization between various EEG rhythms and the cardiac cycle), auto-coherence (stability of a single waveform such as respiration or HRV patterns), and system resonance.

“A coherent heart rhythm is defined as a relatively harmonic (sine-wave-like) signal with a very narrow, high-amplitude peak in the LF region of the HRV power spectrum with no major peaks in the VLF or HF regions. Coherence is assessed by identifying the maximum peak in the 0.04–0.26 Hz range of the HRV power spectrum, calculating the integral in a window 0.030 Hz wide, centered on the highest peak in that region, and then calculating the total power of the entire spectrum. The coherence ratio is formulated as: (Peak Power/[Total Power – Peak Power])” (14).

### The heart rhythm coherence hypothesis

As discussed above, neurocardiology research has established that heart-brain interactions are remarkably complex. Patterns of baroreceptor afferent activity modulate CNS activity over time periods that range from milliseconds to minutes; that is, not only within a cardiac cycle (Armour and Kember, 2004). The intrinsic cardiac ganglia demonstrate both short- and long-term memory. This affects afferent activity rhythms produced by both mechanical variables (e.g., pressure and HR) that occur over milliseconds (single cycles) and hormonal variables that fluctuate over periods ranging from seconds to minutes (Armour, 2003; Armour and Kember, 2004; Ardell et al., 2009). McCraty proposed the heart rhythm coherence hypothesis which states that the pattern and stability of beat-to-beat heart rate activity encode information over “macroscopic time scales,” which can impact cognitive performance and emotional experience. For a more detailed discussion, see McCraty et al. (2009).

### Increasing vagal afferent traffic

Mechanosensitive neurons (baroreceptors) typically increase their firing rates when the rate of change in the function to which they are tuned increases. Heart rhythm coherence, which is characterized by increased beat-to-beat variability and the rate of heart rate change, increases vagal afferent traffic from the cardiovascular system to the brain. This perspective is supported by the MacKinnon et al. (2013) HEP study, discussed earlier, which showed that resonance frequency breathing increased the amount of HRV, HRV coherence, and N250 amplitude in the HEPs. The authors speculated that resonance frequency breathing may have increased vagal afferent traffic and reduced interference with its transmission through subcortical areas to the cerebral cortex.

There has been increasing interest in treating a wide range of disorders with implanted pacemaker-like devices for stimulating the vagal afferent pathways. The FDA has approved these devices for the treatment of epilepsy and depression, and they have been investigated in treating obesity, anxiety, and Alzheimer's disease (Kosel and Schlaepfer, 2003; Groves and Brown, 2005). Neuroradiology research has established that increases in tonic vagal afferent traffic inhibit thalamic pain pathways traveling from the body to the brain at the level of the spinal cord. This finding may explain why studies have shown vagal afferent stimulation can reduce cluster and migraine headaches (Mauskop, 2005) and HRV coherence training reduces chronic pain (Berry et al., 2014).

## Resonance frequency breathing

Lehrer et al.'s resonance frequency model proposes that the delay in the baroreflex system's feedback loops creates each individual's unique cardiovascular system resonance frequency (Lehrer, 2013). While their theoretical model assumes that taller individuals and men have lower resonance frequencies than women and shorter individuals due to the former's larger blood volumes, height only accounts for 30% of the variance in resonance frequency. Breathing, rhythmic muscle tension, and emotional stimulation at a person's resonance frequency can activate or stimulate the cardiovascular system's resonance properties (Lehrer et al., 2009).

They suggest that when people breathe at this rate, which varies in adults from 4.5 to 6.5 breaths per minute, they “exercise” the baroreflex. They have shown that during this paced period, HR and BP oscillations are 180° out of phase, and HRV amplitude is maximized (deBoer et al., 1987; Vaschillo et al., 2002). They also suggest that this phase relationship between HR, respiration, and BP results in the most efficient gas exchange and oxygen saturation (Bernardi et al., 2001; Vaschillo et al., 2004; Yasuma and Hayano, 2004).

With practice, people can learn to breathe at their cardiovascular system's resonance frequency. This aligns the three oscillators (baroreflex, HR, and BP) at that frequency and moves the peak frequency from the HF range (≈0.2 Hz) to the LF range (≈0.1 Hz). Breathing at the resonance frequency more than doubles the energy in the LF band (0.04–0.15 Hz). This corresponds to the Institute of HeartMath's heart rhythm coherence, which is associated with a “narrow, high-amplitude, easily visualized peak” from 0.09 to 0.14 Hz (McCraty et al., 2009; Ginsberg et al., 2010, p. 54).

Resonance frequency breathing is typically used in the context of HRVB training. Several months of steady practice can reset the baroreflex gain so that it is sustained, even when clients are not receiving feedback (Lehrer et al., 2003; Lehrer, 2013). Increased baroreflex gain is analogous to a more sensitive thermostat, allowing the body to regulate BP and gas exchange more effectively (Lehrer, 2007).

## An integrative perspective

There has been a paradigm shift in the medical treatment of diverse disorders like depression, epilepsy, and pain using vagal nerve stimulation (Kosel and Schlaepfer, 2003; Groves and Brown, 2005; Mauskop, 2005). Instead of exclusively targeting sympathetic activation, physicians also attempt to increase vagal tone. Behavioral interventions like HRVB and emotional self-regulation strategies represent non-invasive methods of restoring homeostasis.

HRVB exercises the baroreceptor reflex to enhance homeostatic regulation. Both the heart rhythm coherence and resonance frequency approaches to HRVB teach clients to produce auto-coherent (sinusoidal) heart rhythms with a single peak in the LF region and no significant peaks in the VLF and HF regions (McCraty and Childre, 2010; Lehrer et al., 2013). The coherence model and HEP research (MacKinnon et al., 2013) predict that increased HRV will increase vagal afferent transmission to the forebrain, activate the prefrontal cortex, and improve executive function.

Emotional self-regulation strategies (Forman et al., 2007; McCraty and Atkinson, 2012) can contribute to improved client health and performance, alone, or in combination with HRVB training. McCraty theorizes that emotional self-regulation can increase resilience and accelerate recovery from stressors. From Porges' (2011) perspective, self-regulation through social engagement and bonding can reduce SNS activation while increasing HRV. The CAN model (Thayer et al., 2012) predicts that perception of safety will reduce the activation of the amygdala and increase the prefrontal cortex's ability to exercise top-down control of emotional responses. Finally, from a heart rhythm coherence perspective, emotional self-regulation reduces the SNS activation and/or vagal withdrawal that increase short-term VLF power (Bernardi et al., 1996), decrease shorter-term LF power, and disrupt heart rhythm coherence.

## Summary

The SA node normally generates the heartbeat, which is modulated by autonomic efferent neurons and circulating hormones. There is a dynamic balance between sympathetic and parasympathetic nervous outflows in a healthy, resilient, and responsive nervous system. HRV is generated by multiple regulatory mechanisms that operate on different time scales. Recent findings demonstrate the importance of the intrinsic cardiac nervous system and cardiac afferents in generating the heart rhythm and modulating the time interval between heartbeats. Vagally-mediated HRV appears to represent an index of self-regulatory control, such that individuals with greater resting HRV perform better on tests of executive functions.

Since the LF band primarily reflects the vagally-mediated transmission between the heart and the central nervous system in the context of short-term BP regulation, resting measurements should not be used as markers of SNS activity. Based on 24-h monitoring, ULF and VLF rhythms are more strongly associated with overall health status than HF rhythms. When age-adjusted values are low, they are also more strongly associated with future health risk and all-cause mortality.

HRVB exercises the baroreceptor reflex to enhance homeostatic regulation and restore regulatory capacity. Both the heart rhythm coherence and resonance frequency approaches to HRVB train clients to produce auto-coherent heart rhythms with a single peak in the LF region (typically around 0.1 Hz) and no significant peaks in the VLF and HF regions. Emotional self-regulation strategies can contribute to improved client health and performance, alone, or in combination with HRVB training. A coherent heart is not a metronome since its rhythms are characterized by dynamic complexity with stability over longer time scales.

### Conflict of interest statement

Neither Dr. Fred Shaffer nor Mr. Christopher L. Zerr have any relevant affiliation or financial involvement with any organization or entity with a financial interest or financial conflict with the subject matter discussed in the manuscript. Dr. Rollin McCraty is the Chief Scientist for the Institute of HeartMath, which has generously contributed several of the graphics used in this manuscript.
